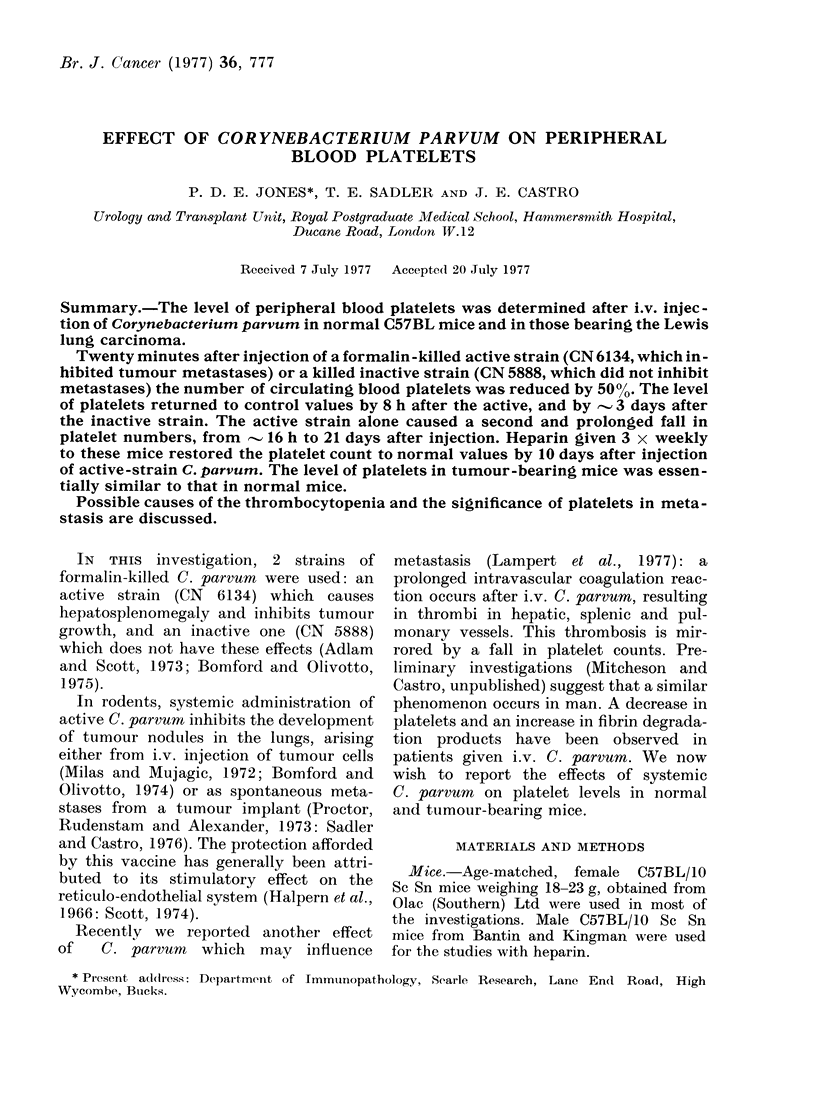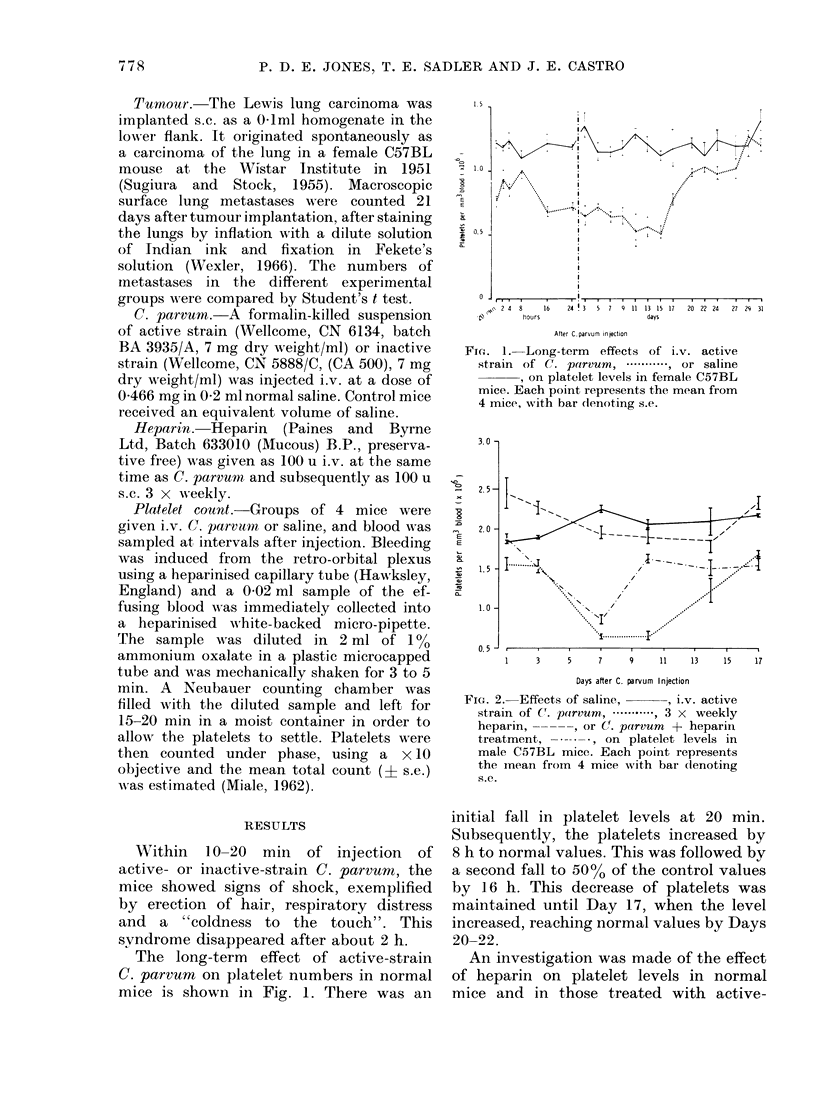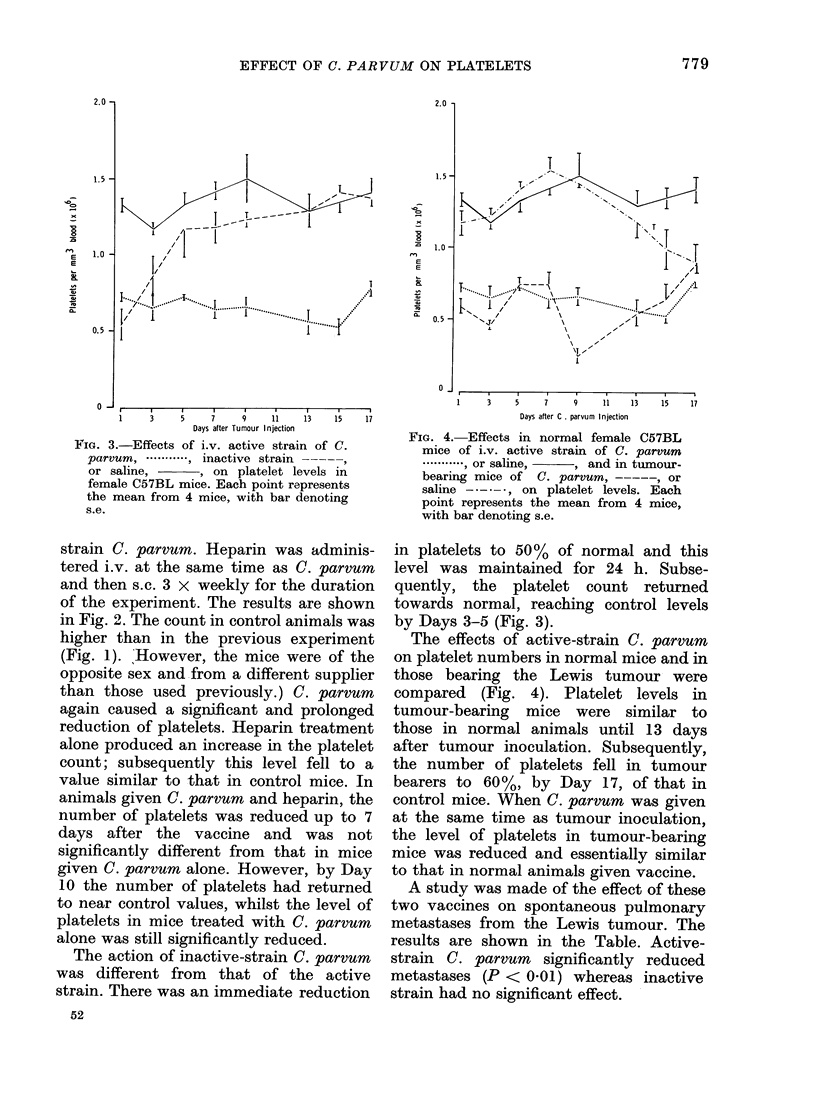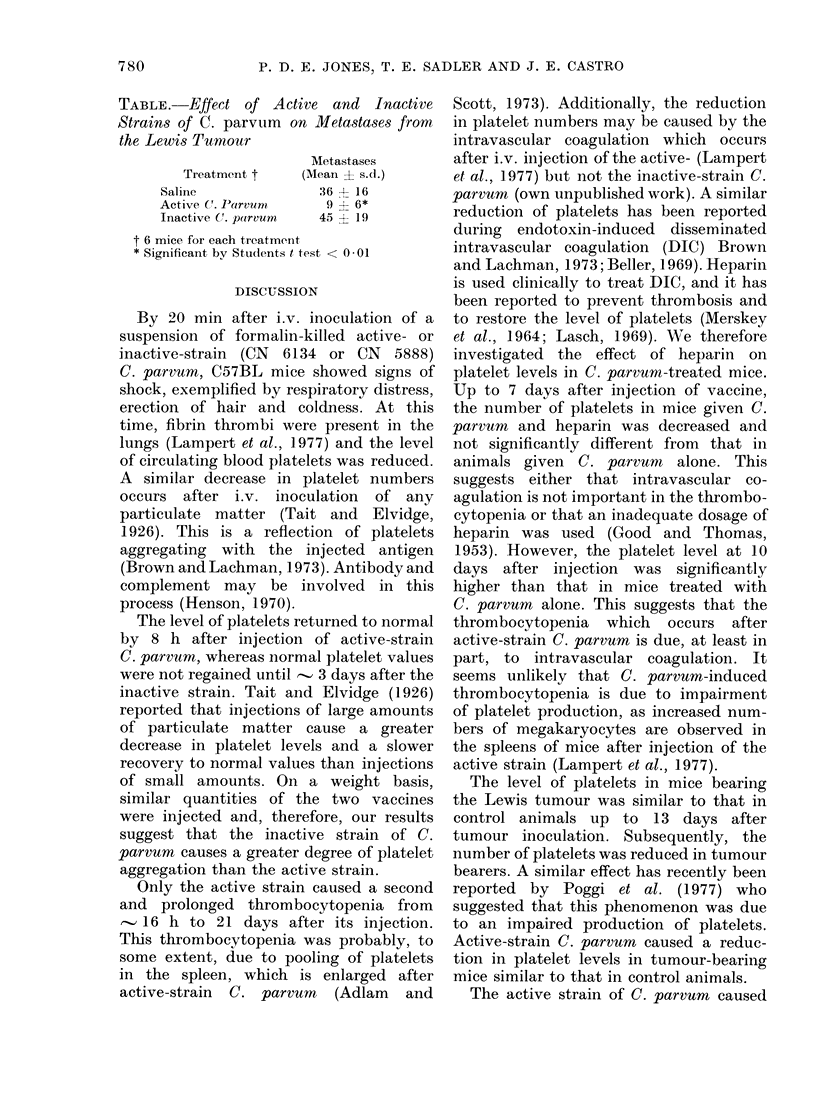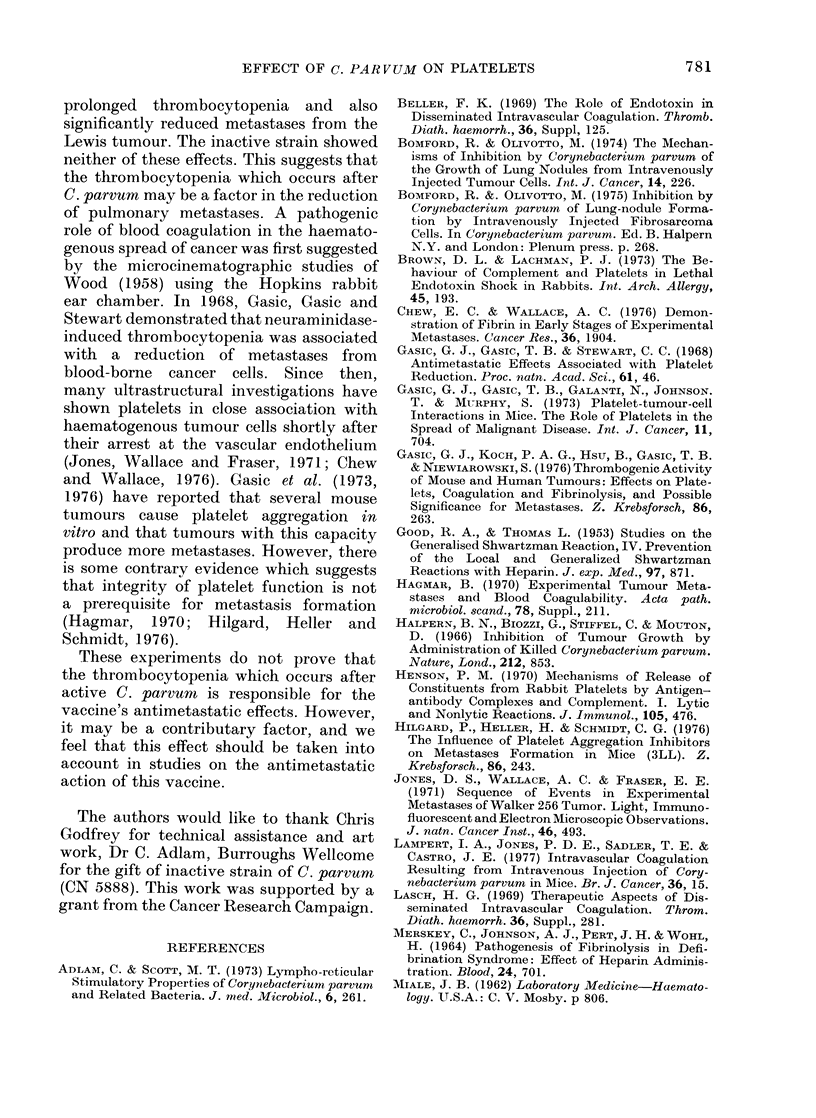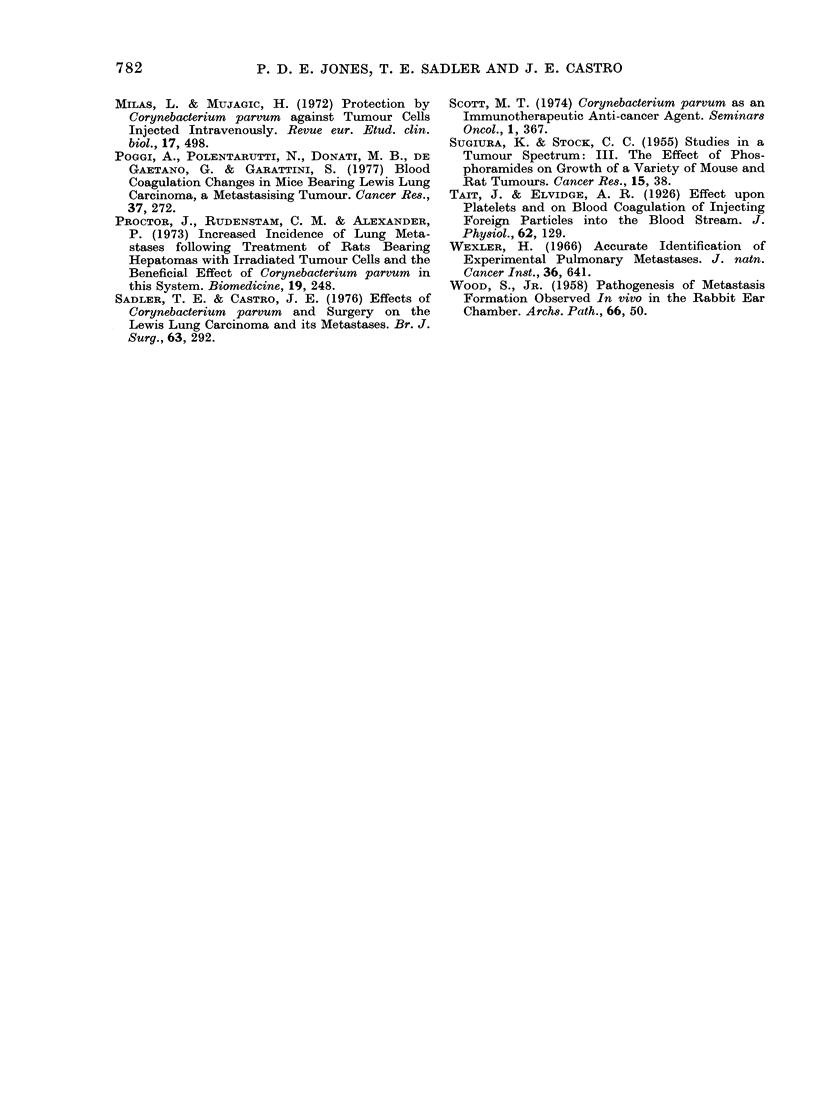# Effect of Corynebacterium parvum on peripheral blood platelets.

**DOI:** 10.1038/bjc.1977.261

**Published:** 1977-12

**Authors:** P. D. Jones, T. E. Sadler, J. E. Castro

## Abstract

The level of peripheral blood platelets was determined after i.v. injection of Corynebacterium parvum in normal C57BL mice and in those bearing the Lewis lung carcinoma. Twenty minutes after injection of a formalin-killed active strain (CN6134, (CN6134, which inhibited tumour metastases) or a killed inactive strain (CN 5888, which did not inhibit metastases) the number of circulating blood platelets was reduced by 50%. The level of platelets returned to control values by 8 h after the active, and by approximately 3 days after the inactive strain. The active strain alone caused a second and prolonged fall in platelet numbers, from approximately 16 h to 21 days after injection. Heparin given 3 X weekly to these mice restored the platelet count to normal values by 10 days after injection of active-strain C. parvum. The level of platelets in tumour-bearing mice was essentially similar to that in normal mice. Possible causes of the thrombocytopenia and the significance of platelets in metastasis are discussed.


*Br. J. Cancer* (1977) **36**, 777

### EFFECT OF *CORYNEBACTERIUM PARVUM* ON PERIPHERAL BLOOD PLATELETS

P. D. E. JONES*, T. E. SADLER AND J. E. CASTRO

*Urology and Transplant Unit, Royal Postgraduate Medical School, Hammersmith Hospital, Ducane Road, London W.12*

Received 7 July 1977 Accepted 20 July 1977

**Summary.—The level of peripheral blood platelets was determined after i.v. injection of *Corynebacterium parvum* in normal C57BL mice and in those bearing the Lewis lung carcinoma.**

**Twenty minutes after injection of a formalin-killed active strain (CN 6134, which inhibited tumour metastases) or a killed inactive strain (CN 5888, which did not inhibit metastases) the number of circulating blood platelets was reduced by 50%. The level of platelets returned to control values by 8 h after the active, and by ~3 days after the inactive strain. The active strain alone caused a second and prolonged fall in platelet numbers, from ~16 h to 21 days after injection. Heparin given 3 × weekly to these mice restored the platelet count to normal values by 10 days after injection of active-strain *C. parvum*. The level of platelets in tumour-bearing mice was essentially similar to that in normal mice.**

**Possible causes of the thrombocytopenia and the significance of platelets in metastasis are discussed.**

IN THIS investigation, 2 strains of formalin-killed *C. parvum* were used: an active strain (CN 6134) which causes hepatosplenomegaly and inhibits tumour growth, and an inactive one (CN 5888) which does not have these effects (Adlam and Scott, 1973; Bomford and Olivotto, 1975).

In rodents, systemic administration of active *C. parvum* inhibits the development of tumour nodules in the lungs, arising either from i.v. injection of tumour cells (Milas and Mujagic, 1972; Bomford and Olivotto, 1974) or as spontaneous metastases from a tumour implant (Proctor, Rudenstam and Alexander, 1973: Sadler and Castro, 1976). The protection afforded by this vaccine has generally been attributed to its stimulatory effect on the reticulo-endothelial system (Halpern *et al.*, 1966: Scott, 1974).

Recently we reported another effect of *C. parvum* which may influence metastasis (Lampert *et al.*, 1977): a prolonged intravascular coagulation reaction occurs after i.v. *C. parvum*, resulting in thrombi in hepatic, splenic and pulmonary vessels. This thrombosis is mirrored by a fall in platelet counts. Preliminary investigations (Mitcheson and Castro, unpublished) suggest that a similar phenomenon occurs in man. A decrease in platelets and an increase in fibrin degradation products have been observed in patients given i.v. *C. parvum*. We now wish to report the effects of systemic *C. parvum* on platelet levels in normal and tumour-bearing mice.

### MATERIALS AND METHODS

*Mice.*—Age-matched, female C57BL/10 Sc Sn mice weighing 18–23 g, obtained from Olac (Southern) Ltd were used in most of the investigations. Male C57BL/10 Sc Sn mice from Bantin and Kingman were used for the studies with heparin.

* Present address: Department of Immunopathology, Searle Research, Lane End Road, High Wycombe, Bucks.

*Tumour*.—The Lewis lung carcinoma was implanted s.c. as a 0·1ml homogenate in the lower flank. It originated spontaneously as a carcinoma of the lung in a female C57BL mouse at the Wistar Institute in 1951 (Sugiura and Stock, 1955). Macroscopic surface lung metastases were counted 21 days after tumour implantation, after staining the lungs by inflation with a dilute solution of Indian ink and fixation in Fekete's solution (Wexler, 1966). The numbers of metastases in the different experimental groups were compared by Student's *t* test.

*C. parvum*.—A formalin-killed suspension of active strain (Wellcome, CN 6134, batch BA 3935/A, 7 mg dry weight/ml) or inactive strain (Wellcome, CN 5888/C, (CA 500), 7 mg dry weight/ml) was injected i.v. at a dose of 0·466 mg in 0·2 ml normal saline. Control mice received an equivalent volume of saline.

*Heparin*.—Heparin (Paines and Byrne Ltd, Batch 633010 (Mucous) B.P., preservative free) was given as 100 u i.v. at the same time as *C. parvum* and subsequently as 100 u s.c. 3 × weekly.

*Platelet count*.—Groups of 4 mice were given i.v. *C. parvum* or saline, and blood was sampled at intervals after injection. Bleeding was induced from the retro-orbital plexus using a heparinised capillary tube (Hawksley, England) and a 0·02 ml sample of the effusing blood was immediately collected into a heparinised white-backed micro-pipette. The sample was diluted in 2 ml of 1% ammonium oxalate in a plastic microcapped tube and was mechanically shaken for 3 to 5 min. A Neubauer counting chamber was filled with the diluted sample and left for 15–20 min in a moist container in order to allow the platelets to settle. Platelets were then counted under phase, using a ×10 objective and the mean total count (± s.e.) was estimated (Miale, 1962).

FIG. 1.—Long-term effects of i.v. active strain of *C. parvum*, ··········, or saline ———, on platelet levels in female C57BL mice. Each point represents the mean from 4 mice, with bar denoting s.e.

FIG. 2.—Effects of saline, ———, i.v. active strain of *C. parvum*, ··········, 3 × weekly heparin, – – – – –, or *C. parvum* + heparin treatment, –·–·–·, on platelet levels in male C57BL mice. Each point represents the mean from 4 mice with bar denoting s.e.

### RESULTS

Within 10–20 min of injection of active- or inactive-strain *C. parvum*, the mice showed signs of shock, exemplified by erection of hair, respiratory distress and a "coldness to the touch". This syndrome disappeared after about 2 h.

The long-term effect of active-strain *C. parvum* on platelet numbers in normal mice is shown in Fig. 1. There was an initial fall in platelet levels at 20 min. Subsequently, the platelets increased by 8 h to normal values. This was followed by a second fall to 50% of the control values by 16 h. This decrease of platelets was maintained until Day 17, when the level increased, reaching normal values by Days 20–22.

An investigation was made of the effect of heparin on platelet levels in normal mice and in those treated with active-

FIG. 3.—Effects of i.v. active strain of *C. parvum*, ··········, inactive strain ––––––, or saline, ————, on platelet levels in female C57BL mice. Each point represents the mean from 4 mice, with bar denoting s.e.

FIG. 4.—Effects in normal female C57BL mice of i.v. active strain of *C. parvum* ··········, or saline, ————, and in tumour-bearing mice of *C. parvum*, ––––––, or saline –·–·–·, on platelet levels. Each point represents the mean from 4 mice, with bar denoting s.e.

strain *C. parvum*. Heparin was administered i.v. at the same time as *C. parvum* and then s.c. 3 × weekly for the duration of the experiment. The results are shown in Fig. 2. The count in control animals was higher than in the previous experiment (Fig. 1). (However, the mice were of the opposite sex and from a different supplier than those used previously.) *C. parvum* again caused a significant and prolonged reduction of platelets. Heparin treatment alone produced an increase in the platelet count; subsequently this level fell to a value similar to that in control mice. In animals given *C. parvum* and heparin, the number of platelets was reduced up to 7 days after the vaccine and was not significantly different from that in mice given *C. parvum* alone. However, by Day 10 the number of platelets had returned to near control values, whilst the level of platelets in mice treated with *C. parvum* alone was still significantly reduced.

The action of inactive-strain *C. parvum* was different from that of the active strain. There was an immediate reduction in platelets to 50% of normal and this level was maintained for 24 h. Subsequently, the platelet count returned towards normal, reaching control levels by Days 3–5 (Fig. 3).

The effects of active-strain *C. parvum* on platelet numbers in normal mice and in those bearing the Lewis tumour were compared (Fig. 4). Platelet levels in tumour-bearing mice were similar to those in normal animals until 13 days after tumour inoculation. Subsequently, the number of platelets fell in tumour bearers to 60%, by Day 17, of that in control mice. When *C. parvum* was given at the same time as tumour inoculation, the level of platelets in tumour-bearing mice was reduced and essentially similar to that in normal animals given vaccine.

A study was made of the effect of these two vaccines on spontaneous pulmonary metastases from the Lewis tumour. The results are shown in the Table. Active-strain *C. parvum* significantly reduced metastases ($P < 0{\cdot}01$) whereas inactive strain had no significant effect.

TABLE.—*Effect of Active and Inactive Strains of* C. parvum *on Metastases from the Lewis Tumour*

| Treatment † | Metastases (Mean ± s.d.) |
| --- | --- |
| Saline | 36 ± 16 |
| Active *C. Parvum* | 9 ± 6* |
| Inactive *C. parvum* | 45 ± 19 |

† 6 mice for each treatment
* Significant by Students *t* test $< 0 \cdot 01$

### DISCUSSION

By 20 min after i.v. inoculation of a suspension of formalin-killed active- or inactive-strain (CN 6134 or CN 5888) *C. parvum*, C57BL mice showed signs of shock, exemplified by respiratory distress, erection of hair and coldness. At this time, fibrin thrombi were present in the lungs (Lampert *et al.*, 1977) and the level of circulating blood platelets was reduced. A similar decrease in platelet numbers occurs after i.v. inoculation of any particulate matter (Tait and Elvidge, 1926). This is a reflection of platelets aggregating with the injected antigen (Brown and Lachman, 1973). Antibody and complement may be involved in this process (Henson, 1970).

The level of platelets returned to normal by 8 h after injection of active-strain *C. parvum*, whereas normal platelet values were not regained until ~ 3 days after the inactive strain. Tait and Elvidge (1926) reported that injections of large amounts of particulate matter cause a greater decrease in platelet levels and a slower recovery to normal values than injections of small amounts. On a weight basis, similar quantities of the two vaccines were injected and, therefore, our results suggest that the inactive strain of *C. parvum* causes a greater degree of platelet aggregation than the active strain.

Only the active strain caused a second and prolonged thrombocytopenia from ~ 16 h to 21 days after its injection. This thrombocytopenia was probably, to some extent, due to pooling of platelets in the spleen, which is enlarged after active-strain *C. parvum* (Adlam and Scott, 1973). Additionally, the reduction in platelet numbers may be caused by the intravascular coagulation which occurs after i.v. injection of the active- (Lampert *et al.*, 1977) but not the inactive-strain *C. parvum* (own unpublished work). A similar reduction of platelets has been reported during endotoxin-induced disseminated intravascular coagulation (DIC) Brown and Lachman, 1973; Beller, 1969). Heparin is used clinically to treat DIC, and it has been reported to prevent thrombosis and to restore the level of platelets (Merskey *et al.*, 1964; Lasch, 1969). We therefore investigated the effect of heparin on platelet levels in *C. parvum*-treated mice. Up to 7 days after injection of vaccine, the number of platelets in mice given *C. parvum* and heparin was decreased and not significantly different from that in animals given *C. parvum* alone. This suggests either that intravascular coagulation is not important in the thrombocytopenia or that an inadequate dosage of heparin was used (Good and Thomas, 1953). However, the platelet level at 10 days after injection was significantly higher than that in mice treated with *C. parvum* alone. This suggests that the thrombocytopenia which occurs after active-strain *C. parvum* is due, at least in part, to intravascular coagulation. It seems unlikely that *C. parvum*-induced thrombocytopenia is due to impairment of platelet production, as increased numbers of megakaryocytes are observed in the spleens of mice after injection of the active strain (Lampert *et al.*, 1977).

The level of platelets in mice bearing the Lewis tumour was similar to that in control animals up to 13 days after tumour inoculation. Subsequently, the number of platelets was reduced in tumour bearers. A similar effect has recently been reported by Poggi *et al.* (1977) who suggested that this phenomenon was due to an impaired production of platelets. Active-strain *C. parvum* caused a reduction in platelet levels in tumour-bearing mice similar to that in control animals.

The active strain of *C. parvum* caused prolonged thrombocytopenia and also significantly reduced metastases from the Lewis tumour. The inactive strain showed neither of these effects. This suggests that the thrombocytopenia which occurs after *C. parvum* may be a factor in the reduction of pulmonary metastases. A pathogenic role of blood coagulation in the haematogenous spread of cancer was first suggested by the microcinematographic studies of Wood (1958) using the Hopkins rabbit ear chamber. In 1968, Gasic, Gasic and Stewart demonstrated that neuraminidase-induced thrombocytopenia was associated with a reduction of metastases from blood-borne cancer cells. Since then, many ultrastructural investigations have shown platelets in close association with haematogenous tumour cells shortly after their arrest at the vascular endothelium (Jones, Wallace and Fraser, 1971; Chew and Wallace, 1976). Gasic *et al.* (1973, 1976) have reported that several mouse tumours cause platelet aggregation *in vitro* and that tumours with this capacity produce more metastases. However, there is some contrary evidence which suggests that integrity of platelet function is not a prerequisite for metastasis formation (Hagmar, 1970; Hilgard, Heller and Schmidt, 1976).

These experiments do not prove that the thrombocytopenia which occurs after active *C. parvum* is responsible for the vaccine's antimetastatic effects. However, it may be a contributary factor, and we feel that this effect should be taken into account in studies on the antimetastatic action of this vaccine.

The authors would like to thank Chris Godfrey for technical assistance and art work, Dr C. Adlam, Burroughs Wellcome for the gift of inactive strain of *C. parvum* (CN 5888). This work was supported by a grant from the Cancer Research Campaign.

### REFERENCES

ADLAM, C. & SCOTT, M. T. (1973) Lympho-reticular Stimulatory Properties of *Corynebacterium parvum* and Related Bacteria. *J. med. Microbiol.*, **6**, 261.

BELLER, F. K. (1969) The Role of Endotoxin in Disseminated Intravascular Coagulation. *Thromb. Diath. haemorrh.*, **36**, Suppl, 125.

BOMFORD, R. & OLIVOTTO, M. (1974) The Mechanisms of Inhibition by *Corynebacterium parvum* of the Growth of Lung Nodules from Intravenously Injected Tumour Cells. *Int. J. Cancer*, **14**, 226.

BOMFORD, R. &. OLIVOTTO, M. (1975) Inhibition by *Corynebacterium parvum* of Lung-nodule Formation by Intravenously Injected Fibrosarcoma Cells. In *Corynebacterium parvum*. Ed. B. Halpern N.Y. and London: Plenum press. p. 268.

BROWN, D. L. & LACHMAN, P. J. (1973) The Behaviour of Complement and Platelets in Lethal Endotoxin Shock in Rabbits. *Int. Arch. Allergy*, **45**, 193.

CHEW, E. C. & WALLACE, A. C. (1976) Demonstration of Fibrin in Early Stages of Experimental Metastases. *Cancer Res.*, **36**, 1904.

GASIC, G. J., GASIC, T. B. & STEWART, C. C. (1968) Antimetastatic Effects Associated with Platelet Reduction. *Proc. natn. Acad. Sci.*, **61**, 46.

GASIC, G. J., GASIC, T. B., GALANTI, N., JOHNSON. T. & MURPHY, S. (1973) Platelet-tumour-cell Interactions in Mice. The Role of Platelets in the Spread of Malignant Disease. *Int. J. Cancer*, **11**, 704.

GASIC, G. J., KOCH, P. A. G., HSU, B., GASIC, T. B. & NIEWIAROWSKI, S. (1976) Thrombogenic Activity of Mouse and Human Tumours: Effects on Platelets, Coagulation and Fibrinolysis, and Possible Significance for Metastases. *Z. Krebsforsch*, **86**, 263.

GOOD, R. A., & THOMAS L. (1953) Studies on the Generalised Shwartzman Reaction, IV. Prevention of the Local and Generalized Shwartzman Reactions with Heparin. *J. exp. Med.*, **97**, 871.

HAGMAR, B. (1970) Experimental Tumour Metastases and Blood Coagulability. *Acta path. microbiol. scand.*, **78**, Suppl., 211.

HALPERN, B. N., BIOZZI, G., STIFFEL, C. & MOUTON, D. (1966) Inhibition of Tumour Growth by Administration of Killed *Corynebacterium parvum*. *Nature, Lond.*, **212**, 853.

HENSON, P. M. (1970) Mechanisms of Release of Constituents from Rabbit Platelets by Antigen–antibody Complexes and Complement. I. Lytic and Nonlytic Reactions. *J. Immunol.*, **105**, 476.

HILGARD, P., HELLER, H. & SCHMIDT, C. G. (1976) The Influence of Platelet Aggregation Inhibitors on Metastases Formation in Mice (3LL). *Z. Krebsforsch.*, **86**, 243.

JONES, D. S., WALLACE, A. C. & FRASER, E. E. (1971) Sequence of Events in Experimental Metastases of Walker 256 Tumor. Light, Immunofluorescent and Electron Microscopic Observations. *J. natn. Cancer Inst.*, **46**, 493.

LAMPERT, I. A., JONES, P. D. E., SADLER, T. E. & CASTRO, J. E. (1977) Intravascular Coagulation Resulting from Intravenous Injection of *Corynebacterium parvum* in Mice. *Br. J. Cancer*, **36**, 15.

LASCH, H. G. (1969) Therapeutic Aspects of Disseminated Intravascular Coagulation. *Throm. Diath. haemorrh.* **36**, Suppl., 281.

MERSKEY, C., JOHNSON, A. J., PERT, J. H. & WOHL, H. (1964) Pathogenesis of Fibrinolysis in Defibrination Syndrome: Effect of Heparin Administration. *Blood*, **24**, 701.

MIALE, J. B. (1962) *Laboratory Medicine—Haematology*. U.S.A.: C. V. Mosby. p 806.

MILAS, L. & MUJAGIC, H. (1972) Protection by *Corynebacterium parvum* against Tumour Cells Injected Intravenously. *Revue eur. Etud. clin. biol.*, **17**, 498.

POGGI, A., POLENTARUTTI, N., DONATI, M. B., DE GAETANO, G. & GARATTINI, S. (1977) Blood Coagulation Changes in Mice Bearing Lewis Lung Carcinoma, a Metastasising Tumour. *Cancer Res.*, **37**, 272.

PROCTOR, J., RUDENSTAM, C. M. & ALEXANDER, P. (1973) Increased Incidence of Lung Metastases following Treatment of Rats Bearing Hepatomas with Irradiated Tumour Cells and the Beneficial Effect of *Corynebacterium parvum* in this System. *Biomedicine*, **19**, 248.

SADLER, T. E. & CASTRO, J. E. (1976) Effects of *Corynebacterium parvum* and Surgery on the Lewis Lung Carcinoma and its Metastases. *Br. J. Surg.*, **63**, 292.

SCOTT, M. T. (1974) *Corynebacterium parvum* as an Immunotherapeutic Anti-cancer Agent. *Seminars Oncol.*, **1**, 367.

SUGIURA, K. & STOCK, C. C. (1955) Studies in a Tumour Spectrum: III. The Effect of Phosphoramides on Growth of a Variety of Mouse and Rat Tumours. *Cancer Res.*, **15**, 38.

TAIT, J. & ELVIDGE, A. R. (1926) Effect upon Platelets and on Blood Coagulation of Injecting Foreign Particles into the Blood Stream. *J. Physiol.*, **62**, 129.

WEXLER, H. (1966) Accurate Identification of Experimental Pulmonary Metastases. *J. natn. Cancer Inst.*, **36**, 641.

WOOD, S., JR. (1958) Pathogenesis of Metastasis Formation Observed *In vivo* in the Rabbit Ear Chamber. *Archs. Path.*, **66**, 50.